# Characterisation of Omicron Variant during COVID-19 Pandemic and the Impact of Vaccination, Transmission Rate, Mortality, and Reinfection in South Africa, Germany, and Brazil

**DOI:** 10.3390/biotech11020012

**Published:** 2022-04-26

**Authors:** Carolina Ribeiro Xavier, Rafael Sachetto Oliveira, Vinícius da Fonseca Vieira, Marcelo Lobosco, Rodrigo Weber dos Santos

**Affiliations:** 1 Department of Computer Science, Federal University of São João del-Rei, São João del-Rei 36301-360, MG, Brazil; carolinaxavier@ufsj.edu.br (C.R.X.); sachetto@ufsj.edu.br (R.S.O.); vinicius@ufsj.edu.br (V.d.F.V.); 2 Department of Computer Science, Federal University of Juiz de Fora, Juiz de Fora 36036-330, MG, Brazil; marcelo.lobosco@ufjf.edu.br

**Keywords:** COVID-19, SIRD, computational epidemiology, vaccination, Omicron variant

## Abstract

Several variants of SARS-CoV-2 have been identified in different parts of the world, including Gamma, detected in Brazil, Delta, detected in India, and the recent Omicron variant, detected in South Africa. The emergence of a new variant is a cause of great concern. This work considers an extended version of an SIRD model capable of incorporating the effects of vaccination, time-dependent transmissibility rates, mortality, and even potential reinfections during the pandemic. We use this model to characterise the Omicron wave in Brazil, South Africa, and Germany. During Omicron, the transmissibility increased by five for Brazil and Germany and eight for South Africa, whereas the estimated mortality was reduced by three-fold. We estimated that the reported cases accounted for less than 25% of the actual cases during Omicron. The mortality among the nonvaccinated population in these countries is, on average, three to four times higher than the mortality among the fully vaccinated. Finally, we could only reproduce the observed dynamics after introducing a new parameter that accounts for the percentage of the population that can be reinfected. Reinfection was as high as 40% in South Africa, which has only 29% of its population fully vaccinated and as low as 13% in Brazil, which has over 70% and 80% of its population fully vaccinated and with at least one dose, respectively. The calibrated models were able to estimate essential features of the complex virus and vaccination dynamics and stand as valuable tools for quantifying the impact of protocols and decisions in different populations.

## 1. Introduction

The first case of Corona Virus Disease (COVID-19) was registered in Wuhan, China, in December 2019. Quickly, the fast spread of the virus in the Chinese city was characterised as an epidemic, and in February 2020, eight countries had already reported cases of the disease. Deeply concerned both by the alarming levels of spread, severity of the disease, and the alarming levels of inaction, the World Health Organisation (WHO) declared COVID-19 as a global pandemic in March 2020 [1].

Until 15 February 2022, COVID-19 confirmed cases reached more than 413.29 million globally, 27.68, 3.65, and 12.69 million in Brazil, South Africa, and Germany, respectively. The global number of COVID-19 deaths, by 15 February 2022, reached more than 5.84 million in the world; 640,076, 97,431, and 120,277 in Brazil, South Africa, and Germany, respectively [2].

**Figure 1:**
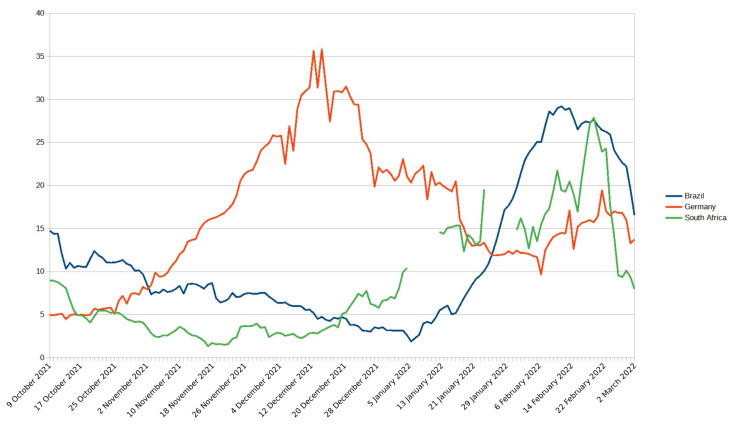
Weekly deaths per million people in recent since 9 October 2021 in South Africa, Germany, and Brazil. Each point represents the cumulative number of confirmed deaths over the previous week.

According to Li et al. [3] in a general context, not only for COVID-19, vaccines could have prevented 69 million deaths between 2000 and 2030, showing that vaccination is fundamental to mitigate the effect of infectious diseases. The conclusion of the tests for the first vaccines for COVID-19 took place in December 2020. Even after the approval of vaccines for COVID-19, 4.09 million people died from COVID-19 in the world, 435,196, 72,740, and 93,877 in Brazil, South Africa, and Germany, respectively, as illustrated in Figure 1.

By 15 February 2022, 4.28 billion people were fully vaccinated with the prescribed initial vaccination protocol, approximately 54% of the global population. Considering the same date, in Brazil, the number of people fully vaccinated is 152.58 million, 71.26% of its population; in South Africa, the number of people fully vaccinated is 17.38 million, 28.95% of its population; and in Germany, the number of people fully vaccinated is 62.32 million people, 74.29% of its population (available at https://ourworldindata.org/covid-vaccinations, accessed on 15 February 2022).

**Figure 2:**
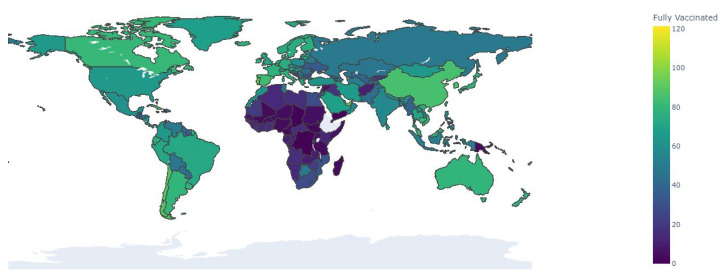
Choropleth map of vaccination around the globe by 15 February 2022. It presents the share of people who received all doses prescribed by the initial vaccination protocol, divided by the total population of the country.

It is known that a rapid vaccination is essential to mitigate the spread of the disease, but the limitations imposed by the productive capacity and a clear definition of a logistics plan for the distribution of the vaccines reduces the potential of application of vaccines in the population, especially in lower-income countries, as illustrated by Figure 2 that present data from [2].

Like any virus, SARS-CoV-2 also mutates. Some of the variants resulting from these mutations may result in a variant of concern, i.e., a new strain that is more infectious, that is more likely to cause new waves or re-infections in those who are previously infected or vaccinated [4,5,6,7]. Five variants of concern, called Alpha [4], Beta [8], Gamma [9], Delta [10,11,12,13], and Omicron [14,15], were identified prior to December 2021.

**Figure 3:**
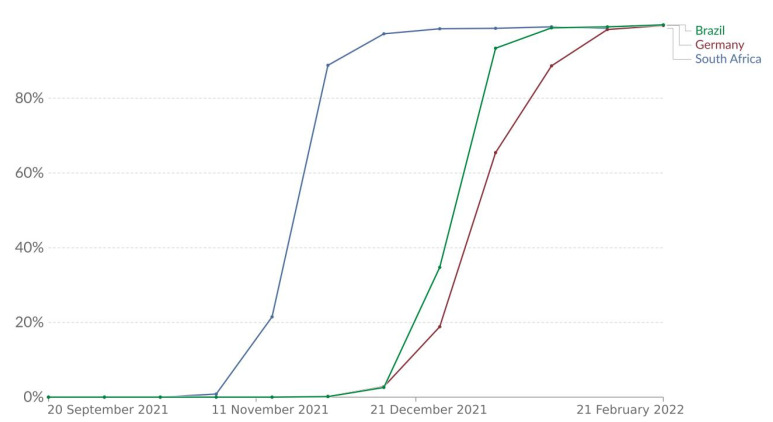
Percentage of Omicron variant of all analysed sequences. This figure was adapted from the one generated by Our World in Data website.

The Omicron variant of the SARS-CoV-2 virus (B.1.1.529) was first detected in South Africa, and it was considered a variant of concern by the World Health Organisation (WHO) on 11 November 2021. Since then, it has been spread worldwide: by mid-January, it was the most predominant strain on the planet, causing a considerable increase in COVID-19 cases. In many countries, the Omicron variant represented a resurgence of the pandemic, disrupting the trend of decreasing numbers of COVID-19 cases and deaths. Figure 3 presents the share of the Omicron variant in all analysed sequences for the studied countries.

Many models have been proposed to describe the dynamics of epidemics. Some of them can be classified into two categories, collective models [16,17,18,19,20,21,22,23] and network-based models [24,25,26,27,28,29]. Some studies have investigated how vaccination and non-pharmacological strategies can impact the course of epidemics [21,22,23,30,31,32,33]. The work presented in [34] investigates the 2009–2010 A(H1N1)pdm09 virus propagation and states that the timing of the vaccination program significantly influences the efficacy of immunisation. Nguyen and Carlson [35] consider a stochastic SIR model to analyse the impact of time delays of vaccination in the epidemics, allowing the authors to define optimal resource allocation strategies. The paper shows that the epidemic is more effectively eradicated, requiring fewer vaccines, when adopting early mass vaccination. Rodrigues et al. [24] use a network-based approach to choose individuals to receive vaccine that minimises the A(H1N1) impact in a hypothetical population.

In [36], the use of a simple mathematical model was proposed, based on the classical SIRD model to adjust and predict the COVID-19 pandemics behaviour in three countries: Brazil, Italy, and Korea, which are examples of very different scenarios and stages of the COVID-19 pandemic. The model used in this work is also based on the classic compartmental SIRD model and extends the models proposed in [37,38,39].

Many works considered different models to try to reproduce, understand and predict the COVID-19 pandemic. Giordano et al. [40] uses the SIDARTHE model (susceptible (S), infected (I), diagnosed (D), ailing (A), recognized (R), threatened (T), healed (H), and extinct (E)) for modelling the COVID-19 epidemic and implementation of population-wide interventions in Italy, considering different levels of severity of the disease. Yang et al. [19] use the SEIR model (susceptible (S), exposed (E), infected (I), and removed (R)) to derive the epidemic curve. They also used artificial intelligence (AI) to predict the epidemic, trained on the SARS data. Li et al. [20] use a Monte Carlo method in the building stage of the model for a forward prediction and a backward inference of the epidemic situation.

The main goal of this work is to characterise the behaviour of the pandemic in three different countries, observing some aspects of the virus’ dynamics before and after the emergence of the Omicron variant. For this purpose, we have extended a mathematical model previously presented in [38]. In particular, to reproduce public epidemiological data in the studied countries, new features were needed in the model: mortality rates previous and after Omicron, transmission rates previous and after Omicron, vaccination efficacy, and the possibility of reinfection.

The remainder of the work is organised as follows: in Section 2, the proposed model is detailed; in Section 3 the results of the simulations are presented; in Section 4 the results are discussed; in Section 5 the limitations of the work are described, and finally, the conclusions of the work are presented in Section 6.

## 2. Material and Methods

### 2.1. Mathematical Model

The SIRD is a simple compartmental model that divides a population into four states. The model parameters are only three rates as presented by the set of equations:

(1) $$\left\{\begin{array}{cc} \frac{dS}{dt} & =-\frac{\alpha_{o}}{N}SI,\\ \frac{dI}{dt} & =\frac{\alpha_{o}}{N}SI-\beta_{o}I-\gamma_{o}I,\\ \frac{dR}{dt} & =\gamma_{o}I,\\ \frac{dD}{dt} & =\beta_{o}I, \end{array}\right.$$

where *S* (susceptible), *I* (infected), *R* (recovered), *D* (dead) are the variables that represent the number of individuals within a population of size *N*. The terms $\alpha_{o}$, $\beta_{o}$, and $\gamma_{o}$ denote infection rates, recovery, and mortality, respectively. The SIRD model is a very used model in the literature and is commonly extended to reproduce different dynamics of the disease behaviour such as vaccination effects, the change in the rates over time, possible underreporting of confirmed cases or other populational behaviour. In our model, we extend the model of [38] that captures these issues that the classical model cannot provide. In addition, we tried to keep the model as simple as possible to reduce the number of unknown parameters to be estimated. The following set of equations describes our model:

(2) $$\left\{\begin{array}{cc} \frac{dS}{dt} & =-\frac{\alpha (t)}{N}SI,\\ \frac{dI}{dt} & =\frac{\alpha (t)}{N}SI-\beta \left(t\right)I-\gamma \left(t\right)I,\\ \frac{dR}{dt} & =\gamma (t)I,\\ \frac{dD}{dt} & =\beta (t)I,\\ I_{r} & =\theta I,\\ R_{r} & =\theta R,\\ C & =I_{r}+R_{r}+D, \end{array}\right.$$

where *S* (susceptible), *I* (infected), *R* (recovered), *D* (dead), $I_{r}$ (reported as infected), $R_{r}$ (reported as recovered), and *C* (total confirmed cases) are the variables that represent the number of individuals within a population of size *N*. The term α(t) denotes the rate at which a susceptible individual becomes infected and it is given by Equation (3):

(3) $$\alpha \left(t\right)=a\left(t,r_{1},t_{i_{1}},t_{f_{1}}\right)a\left(t,r_{2},t_{i_{2}},t_{f_{2}}\right)b,$$

where *b* is the basic transmission rate, and the terms $a_{1}=a\left(t,r_{1},t_{i_{1}},t_{f_{1}}\right)$ and $a_{2}=a\left(t,r_{2},t_{i_{2}},t_{f_{2}}\right)$ represent different stages of the transmission rate. Two different modifications of transmission rates, $r_{1}$ and $r_{2}$, are adopted. In this way, $a_{1}$ accounts for the impact of mitigation policies, such as social distance, before the emergence of Omicron, whereas $a_{2}$ is related to the impact of the Omicron variant in the transmission.

The function $a(t,r,t_{i},t_{f})$ is given by Equation (4):

(4) $$a\left(t,r,t_{i},t_{f}\right)=\left\{\begin{array}{cc} 1, & t<t_{i}\\ \frac{1-r}{t_{i}-t_{f}}\left(t-t_{i}\right)+1, & t_{i}\leq t\;\mathrm{and}\;t\leq t_{f}\\ r, & c.\;c. \end{array}\right.$$

This simple approach used for a(t) assumes that when restriction policies start to be adopted at $t_{i_{1}}$, the probability of contact is multiplied by $r_{1}<1.0$ at the end time $t_{i_{1}}+\Delta_{1}$. As the new variant appears at the time instant $t_{i_{2}}$, transmission factor is multiplied by $r_{2}>1.0$ in the final time ($t_{i_{2}}+\Delta_{2}$).

In this work, we also modified the mortality rate, m(t) according to the vaccination rate and to the arrival of the Omicron variant, where $\beta \left(t\right)=m\left(t\right)/\tau_{0}$. The number of days from infection until death is represented by $\tau_{0}=\tau_{1}+\tau_{2}$, where $\tau_{1}$ is the incubation time of the virus and $\tau_{2}$ is the time between the first symptoms until death. The rate at which infected individuals recover from the virus is given by $\gamma \left(t\right)=\left(1-m\left(t\right)\right)\left(1/\tau_{r}\right)$, where $\tau_{r}$ is the number of days from infection until recovery with $\tau_{r}=\tau_{1}+\tau_{3}$. $\tau_{3}$ is the time between the first symptoms until recovery. The percentage of confirmed infected individuals that are notified or reported is represented by θ.

As mentioned before, m(t) changes according to the vaccination rate and to the arrival of the Omicron variant. The time-dependent parameter m(t) represents a weighted average between the mortality rate among vaccinated v(t) and unvaccinated (1−v(t)) people and the transition from a mortality rate previous to Omicron *m* to a mortality rate during Omicron $r_{d}\;m$, with $r_{d}<1.0$:

(5) $$m\left(t\right)=\left(m\left(1.0-v\left(t\right)\right)\right)+\left(m\left(1.0-efi_{d}\right)v\left(t\right)\right)mo\left(t\right),$$

where v(t) is input data that represent the fraction of the population that is fully vaccinated, $efi_{d}$ is the reduction in mortality among the fully vaccinated population, and mo(t), the mortality during Omicron, follows the same dynamics of $a_{2}$, the transmission rate during Omicron: $mo\left(t\right)=mo\left(t,r_{d},t_{i_{2}},t_{f_{2}}\right)$, where $r_{d}$ is the mortality reduction factor during Omicron.

Therefore, different from the model presented before in [38], here we assume that the impact of vaccination on the transmission rate during Omicron is negligible. The model only accounts for the reduction in mortality among the fully vaccinated population (parameter $efi_{d}<1.0)$.

Finally, with the modifications above, we still could not fit the model to the data. One last hypothesis was needed: the possibility of reinfection. This was modelled by using the following initial condition for the susceptible population: $S\left(t_{0}\right)=total\;population-death\left(t_{0}\right)-recovered\left(t_{0}\right)\left(1-s_{rate}\right)$; therefore, if the parameter $s_{rate}=1$ we have the potential to reinfect all the already recovered population, whereas when $s_{rate}=0$ we have the case of zero reinfections.

### 2.2. Numerical Simulations

The differential evolution (DE) optimisation method [41] was used to estimate each parameter of the mathematical model described in Section 2.1 to publicly available data for Brazil, South Africa, and Germany with the same approach as described before in our previous works [36,37,38]. For this purpose, an in-house implementation was developed using the C programming language.

The objective function consists of the weighted sum of the errors between the active, deaths, and confirmed cases generated by the simulations and the corresponding publicly available data. Here, we consider $\hat{I}\left(t\right)$ as the reported numbers of active cases, $\hat{D}\left(t\right)$ the number of deaths, and $\hat{C}\left(t\right)$ the total confirmed cases. The objective function described by Equation (7), was used to minimise the relative error ($R_{E}\left(\lambda ,\hat{\lambda }\right)$) between the data and the model described by Equation (6):

(6) $$R_{E}\left(\lambda ,\hat{\lambda }\right)=\frac{∥\lambda \left(t,p\right)-\hat{\lambda }{\left(t\right)∥}_{1}}{∥\hat{\lambda }{\left(t\right)∥}_{1}},$$

(7) $$\min_{p}\left(O\left(p\right)=\omega_{1}R_{E}\left(I,\hat{I}\right)+\omega_{2}R_{E}\left(D,\hat{D}\right)+\omega_{3}R_{E}\left(C,\hat{C}\right)\right),$$

where *p* is the set of parameters to be estimated and $\omega_{n}$ is a weight. For this work, we used $\omega_{1}=\omega_{2}=1$ and $\omega_{3}=2.0$ for Brazil and $\omega_{1}=\omega_{2}=\omega_{3}=1.0$ for South Africa and Germany.

Instead of taking only the best fit, we consider the existence of a model discrepancy [42] of 10%; therefore, all the parameters *p* that satisfy O(p)<10% is taken as a viable solution.

### 2.3. Data Sources

The model was calibrated using the data publicly available online [2]. The data considered for the simulation in Brazil and South Africa range from 1 August 2021 and 15 February 2022 (197 days). For Germany, data for simulation range from 26 August 2021 and 15 February 2022 (112 days). The difference in the periods considered for each country is due to the moments that each wave occurred.

## 3. Results

**Figure 4:**
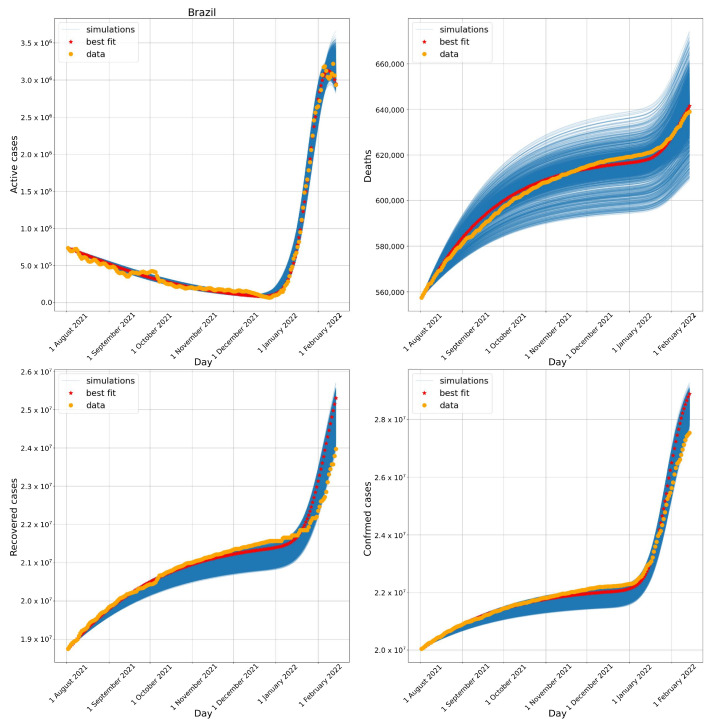
Evolution of the number of active cases (**upper left**), deaths (**upper right**), recovered cases (**lower left**), and confirmed cases (**lower right**) in Brazil. All simulations with errors below 10% are presented.

**Figure 5:**
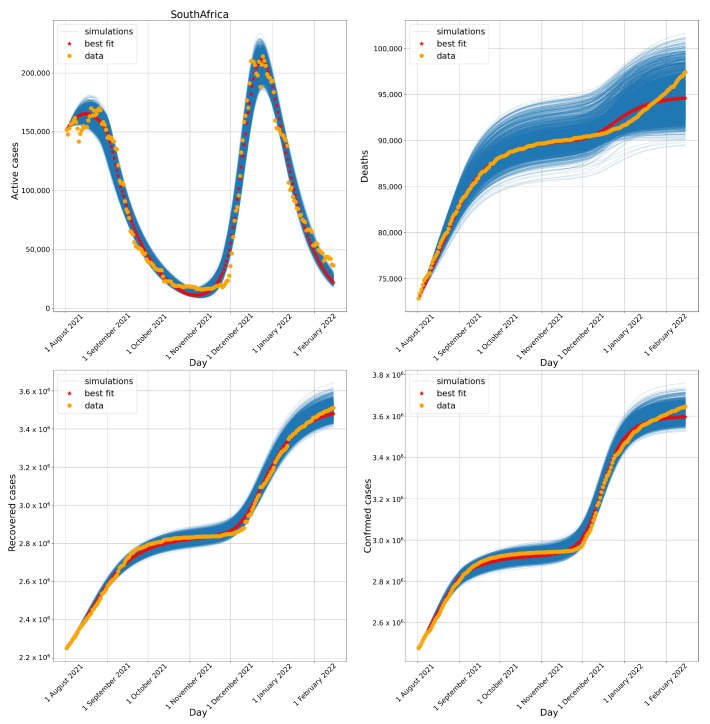
Evolution of the number of active cases (**upper left**), deaths (**upper right**), recovered cases (**lower left**), and confirmed cases (**lower right**) in South Africa. All simulations with errors below 10% are presented.

**Figure 6:**
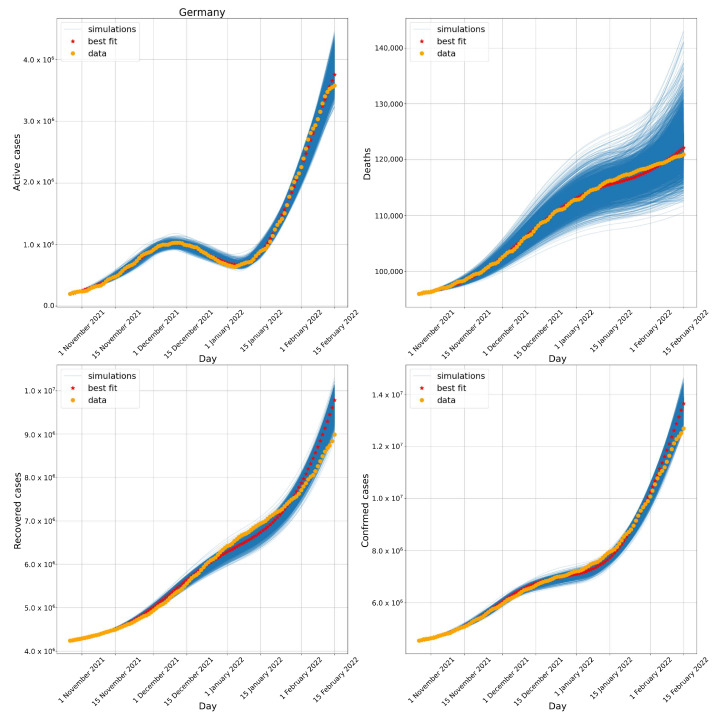
Evolution of the number of active cases (**upper left**), deaths (**upper right**), recovered cases (**lower left**), and confirmed cases (**lower right**) in Germany. All simulations with errors below 10% are presented.

Figure 4, Figure 5 and Figure 6 present the results of the numerical simulations. These three figures illustrate the evolution of active cases, deaths, recovered cases, and confirmed cases in Brazil, South Africa, and Germany, respectively, comparing them to public data reported by these countries. Each figure presents the best fit obtained by the DE algorithm, as well as all DE results whose errors compared to the public dataset were below 10%.

### 3.1. Brazil

Figure 4 compares the number of active cases, deaths, recovered cases, and confirmed cases obtained numerically to the real data available to Brazil. The results show that the model successfully captured the complex behaviour of the pandemic. More specifically, the numerical results reproduce the decay observed between August 2021 to January 2022, followed by a rapid increase in the number of active cases observed between January and February 2022 and the apparent peak achieved in the middle of February. In addition, the model was able to capture similar behaviour observed in the number of deaths, recovered, and confirmed cases.

Adding the first day of simulation for Brazil to the parameters found for the transition phase to Omicron, $ti_{2}$ and $\Delta_{2}$, we arrive at dates very close to those reported in Figure 3. The model calibration suggested that the Omicron variant arrived approximately between mid-December 2021 (19 December 2021) and early January 2022 (10 January 2022). From the data presented in Figure 3, it is possible to observe that, by 27 December 2021, the Omicron variant represented 34% of sequenced cases and by 10 January 2022, 93% of sequenced cases.

### 3.2. South Africa

Figure 5 compares the number of active cases, deaths, recovered cases, and confirmed cases obtained numerically to the data available to South Africa. South Africa represents a huge challenge to the model due to the two waves observed in the active cases data, which also was successfully captured by the numerical results. The model was also successful in capturing the behaviour of the other curves, although a small misfit can be observed after the middle of December 2021 in the number of deaths.

Adding the first day of simulation for South Africa to the parameters found for the transition phase to Omicron, $ti_{2}$ and $\Delta_{2}$, we arrive at dates very close to those reported in Figure 3. The model calibration suggested that the Omicron variant arrived approximately between the end of October 2021 (27 October 2021) and early December 2021 (1 December 2021). From the real data, it is possible to observe that by November 2021 (15 November 2021), the Omicron variant represented 21% of sequenced cases and by 12 December 2021, 97% of sequenced cases.

### 3.3. Germany

Figure 6 compares the number of active cases, deaths, recovered, and confirmed cases obtained numerically to the data available from Germany. Again, the numerical results captured both waves observed between November 2021 and January 2022 and their distinct impacts on the number of deaths.

Adding the first day of simulation for Germany to the parameters found for the transition phase to Omicron, $ti_{2}$ and $\Delta_{2}$, we arrive at dates very close to those reported in Figure 3. The model calibration suggested that the Omicron variant arrived approximately between mid December 2021 (31 December 2022) and early January 2022 (17 January 2022). From the real data, it is possible to observe that by 27 December 2021, the Omicron variant represented 18% of sequenced cases and by 24 January 2022, 89% of sequenced cases.

### 3.4. Estimated Parameters for the Three Different Countries

Table 1 presents the parameters’ values found by the DE algorithm that produce the best fit to each country’s data. These parameters represent the characterisation of the COVID-19 pandemics by country: *b* represents the basic infection rate, *m*, the mortality rate previous to Omicron; $r_{1}$, the contact reduction factor; $t_{i_{1}}$, the start time for intervention policy 1; $\Delta_{1}$, the intervention policy 1 duration; $r_{2}$, Omicron transmission rate factor; $t_{i_{2}}$, the start time for the Omicron variant; $\Delta_{2}$, the transition to Omicron duration; $\tau_{1}$, the incubation period; $\tau_{2}$, the time from symptoms to death; $\tau_{3}$, the time from symptoms to recovery; θ is the notified cases; $eff_{d}$ is the vaccine efficacy for prevent deaths; $s_{rate}$ is the potential of reinfection; rd is the mortality reduction factor during Omicron.

**Figure 7:**
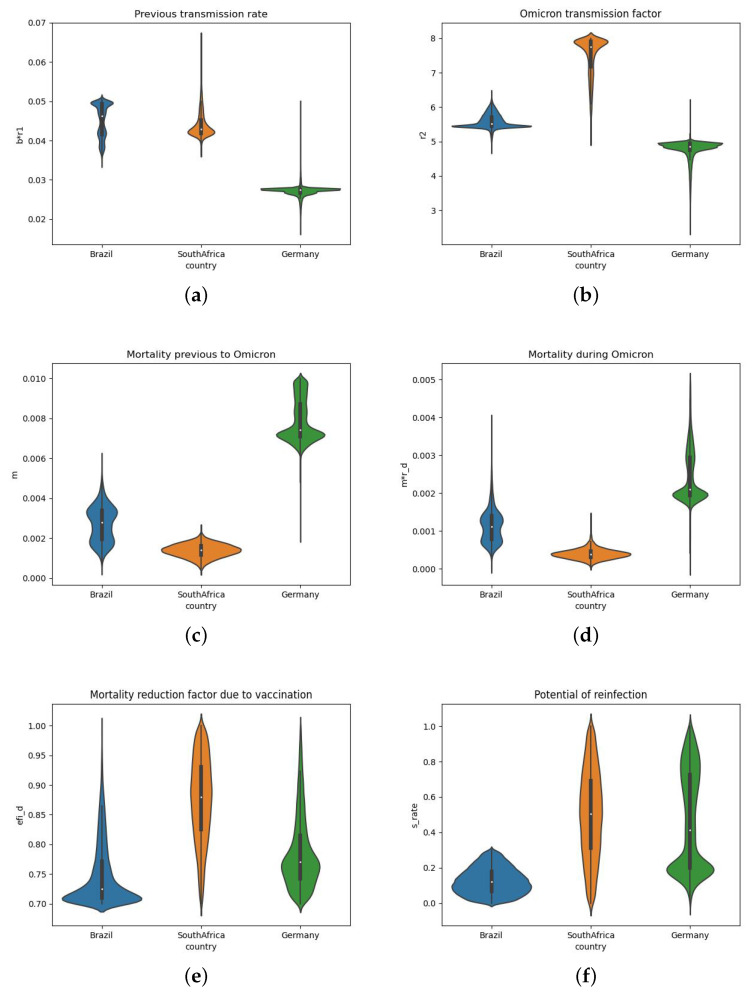
Violin plots of the parameters: br1, r2, *m*, $m\;r_{d}$, $eff_{d}$, $s_{rate}$ for Brazil, South Africa, and Germany. (**a**) br1. (**b**) $r_{2}$. (**c**) *m*. (**d**) mrd. (**e**) $eff_{d}$. (**f**) $s_{rate}$.

The set of parameters found by the DE executions that produced errors below 10% were also analysed. These values are presented in Figure 7 as violin plots, each one with the distribution of the following estimated parameters: br1 (previous transmission rate), Omicron transmission factor (r2), mortality previous to Omicron (*m*), mortality during Omicron ($m\;r_{d}$), mortality reduction factor due to vaccination ($effi_{d}$), and potential of reinfection ($s_{rate}$).

## 4. Discussion

The results presented in Section 3 show that the model was able to adjust their numerical results to real data for Brazil (Figure 4), South Africa (Figure 5), and Germany (Figure 6). Table 1 and Figure 7 compare the three countries. For 95% of the simulations, the transmission rate ($b*r_{1}$) was very low before Omicron, less than 0.05 for Brazil and South Africa, and less than 0.03 for Germany. The lowest value for Germany is in accordance with the more strict mitigation and isolation policies adopted.

During the Omicron wave, the transmission rate was significantly amplified (parameter $r_{2}$) by factors between 5 (for Brazil and Germany) and 8 (for South Africa).

The parameter $\Delta_{2}$ estimates the duration that the Omicron variant took to spread in the studied countries. The values observed for South Africa were the highest among the simulations, standing in the range between 20 and 40, with a mean value of 30. For Germany, the values found are distributed between 15 and 20 with 18 as mean. For Brazil, the observed values for $\Delta_{2}$ stand between 18 and 25. These observations corroborate the prevalence of the Omicron variant in the simulated period, as seen in real data presented in Figure 3.

From the results, we note that the mortality rate before Omicron (*m*) in Germany (0.0074) is higher than in Brazil 0.0022 and in South Africa 0.0016 for the analysed period. This may be related to the Delta variant that has hit Germany and other European countries harder than South Africa and Brazil. In addition, the observed mortality rates are also in accordance with the median age of the population of these countries: 27 years old for South Africa, 33 for Brazil, and 45 for Germany [43].

The mortality rate during Omicron ($m*r_{d}$) decreased significantly in comparison to the previous period, which can be verified by the value of the parameter $r_{d}$. For Brazil, the mortality during Omicron is 41% of the mortality before Omicron; for South Africa, this value is 30%; and for Germany, 28%; therefore, on average, the mortality during Omicron decreased by three-fold. The decrease in severity and mortality for Omicron is pointed out by several studies in different localities as Ontario (Canada) [44], South Africa [45], and England [46].

It is also worth highlighting the results found for the parameter $eff_{d}$, the mortality reduction factor due to vaccination, after the model calibrations. The best values found were all around 70% and 75%. This means that the nonvaccinated population in these countries have, on average, three to four times higher chances of dying due to COVID-19 complications than the fully vaccinated population.

The parameter θ reflects that underreporting has significantly increased during Omicron, which is likely due to the high numbers of infected persons who are asymptomatic or have mild symptoms. For instance, the value of θ, the fraction of cases that are reported, dropped from 0.63 (as previously calibrated for Germany in [37]) to 0.24 during the Omicron phase.

Finally, the values obtained for the parameter $s_{rate}$, which represents the share of people that can be reinfected in each country, is the highest for South Africa (41%), followed by Germany (21%) and Brazil (13%). The high number of reinfections in South Africa is likely due to the poor vaccination coverage (28.95% of its population) compared to the numbers found in Brazil (71.26% of its population) and Germany (74.29%). The small difference between Brazil and Germany may be related to the population with one dose of the vaccine, 76% in Germany against 83% in Brazil.

## 5. Limitations and Future Works

The model considered in this work, as well as the model in which it is based [38], is able to adjust well to real data, as presented in Section 3; however, it shows a series of limitations, which we intend to discuss in this section.

As the first case of COVID-19 occurred not much more than two years ago, some limitations of our model relate to uncertainties regarding the disease’s characteristics, which only recently has been investigated. Currently, there is no consensus on how often reinfections of COVID-19 can occur and how a recurrent occurrence of the disease could affect the dynamics of its transmission. Our initial results suggest that reinfections could be high (20 to 40% of the populations of the analysed countries); however, the lack of data in the public repositories about reinfections prevented us from validating these results. As these data become more readily available and in the case the amount of reinfections predicted by our model is confirmed, SIRS-like models will be more appropriate to study the endemic dynamics of COVID-19.

Other limitations are imposed by the complex behaviour of a population, especially facing a pandemic, due to explicit public policies and guidelines or the self-organisation of people. As the pandemic progresses, people harden or soften social distancing and adopt measures such as masks and hand sanitation; restaurants, schools, and business places for other economic activities close and open as the occupancy of hospitals change. The fine-scale variations of the social dynamics are not considered by our model and represent a limitation of our work.

As reported in Section 2, the model proposed in this work is intended to be kept as simple as possible. The limitations here described do not prevent us from better understanding the pandemic dynamics and further improving our model in future works.

## 6. Conclusions

In this work, we proposed a computational model that was able to reproduce the Omicron wave in different countries: South Africa, Brazil, and Germany. By solving the inverse problem associated with the calibration of the model to the available epidemiological data, we were able to characterise the impact of the Omicron variant in these three countries.

As common features, we were able to verify that the estimated transmission rate of the Omicron wave was five to eight times higher than before the appearance of this variant. On the other hand, after the Omicron variant was established, mortality, on average, decreased by three-fold. Finally, we have observed that underreporting was significant in all three analysed countries. Our results suggest that the reported cases of COVID-19 account for less than 25% of the actual cases during the Omicron wave. This is likely due to a significant number of infected persons who are asymptomatic or have mild symptoms.

Interesting differences between the countries were also observed in this study. For instance, the mortality rates before and during Omicron were much higher in Germany than in Brazil and South Africa. This could be related to the Delta variant that has hit Germany and other European countries harder than South Africa and Brazil. Alternatively, it could be just reflecting the median age of the population of these countries: 27 years old for South Africa, 33 for Brazil, and 45 for Germany.

Our results also highlighted the impact of vaccination on the pandemic dynamics. We observed that the mortality among the nonvaccinated population in these countries is, on average, three to four times higher than the mortality among the fully vaccinated population. Finally, we could only reproduce the observed dynamics after introducing a new parameter in the mathematical model to account for the percentage of the population that can be reinfected. The number of reinfections was between 20 and 40%. In addition, we observed an opposite correlation between reinfections and the vaccination coverage or status of the country. Reinfection was as high as 40% in South Africa, which has only 28.95% of its population fully vaccinated and as low as 13% in Brazil, which has over 70% and 80% of its population fully vaccinated and with at least one dose, respectively. Finally, it is worth mentioning that this simple model could reproduce several complex aspects of the pandemic; therefore, it is a valuable tool for decision makers to understand the dynamics of the virus and the ongoing vaccination protocol as well as their quantitative impact on different populations.

## Figures and Tables

**Table 1:** Characterisation of the COVID-19 pandemics by the best parameter values by country: *b* represents the basic infection rate, *m*, the mortality rate previous to Omicron; $r_{1}$, the contact reduction factor; $t_{i_{1}}$, the start time for intervention policy 1; $\Delta_{1}$, the intervention policy duration; $r_{2}$, Omicron transmission rate factor; $t_{i_{2}}$, the start time for the Omicron variant; $\Delta_{2}$, the transition to Omicron duration; $\tau_{1}$, the incubation period; $\tau_{2}$, the time from symptoms to death; $\tau_{3}$, the time from symptoms to recovery; θ is the notified cases; $eff_{d}$ is the vaccine efficacy for prevent deaths; $s_{rate}$ is the potential of reinfection; rd is the mortality reduction factor during Omicron.

Parameters	Brazil	South Africa	Germany
*b*	0.049745	0.083456	0.098533
*m*	0.002223	0.001614	0.007456
$r_{1}$	0.999733	0.503252	0.279499
$ti_{1}$	37.611357	27.571859	27.361629
$\Delta_{1}$	43.476986	7.465598	25.647412
$r_{2}$	5.422328	7.993557	4.961679
$ti_{2}$	139.978414	86.668599	65.600770
$\Delta_{2}$	21.272066	34.562475	17.164128
$\tau_{1}$	6.305544	4.653837	11.486396
$\tau_{2}$	18.950821	33.184733	27.741349
$\tau_{3}$	11.803360	9.761925	10.689566
θ	0.052279	0.019445	0.241283
$eff_{d}$	0.702603	0.753452	0.761646
$s_{rate}$	0.133930	0.419652	0.211434
rd	0.410348	0.283680	0.278265

## Data Availability

The datasets used for this study can be found in Data on COVID-19 (coronavirus) by Our World in Data at https://github.com/owid/covid-19-data/tree/master/public/data/ [2], accessed on 15 February 2022.

## References

[1] World Health Organization WHO Timeline—COVID-19. 27 April 2020. https://www.who.int/news/item/27-04-2020-who-timeline-covid-19.

[2] Our World in Data Coronavirus Pandemic (COVID-19). https://ourworldindata.org/coronavirus..

[3] Li X., Mukandavire C., Cucunubá Z.M., Londono S.E., Abbas K., Clapham H.E., Jit M., Johnson H.L., Papadopoulos T., Vynnycky E. (2021). Estimating the health impact of vaccination against ten pathogens in 98 low-income and middle-income countries from 2000 to 2030: A modelling study. Lancet.

[4] Davies N.G., Abbott S., Barnard R.C., Jarvis C.I., Kucharski A.J., Munday J.D., Pearson C.A., Russell T.W., Tully D.C., Washburne A.D. (2021). Estimated transmissibility and impact of SARS-CoV-2 lineage B. 1.1. 7 in England. Science.

[5] Ong S.W.X., Chiew C.J., Ang L.W., Mak T.M., Cui L., Toh M.P.H.S., Lim Y.D., Lee P.H., Lee T.H., Chia P.Y. (2021). Clinical and virological features of SARS-CoV-2 variants of concern: A retrospective cohort study comparing B.1.1.7 (Alpha), B.1.315 (Beta), and B.1.617.2 (Delta). Clin. Infect. Dis..

[6] Salleh M.Z., Derrick J.P., Deris Z.Z. (2021). Structural evaluation of the spike glycoprotein variants on SARS-CoV-2 transmission and immune evasion. Int. J. Mol. Sci..

[7] Sanches P.R., Charlie-Silva I., Braz H.L., Bittar C., Calmon M., Rahal P., Cilli E.M. (2021). Recent advances in SARS-CoV-2 Spike protein and RBD mutations comparison between new variants Alpha (B. 1.1. 7, United Kingdom), Beta (B. 1.351, South Africa), Gamma (P. 1, Brazil) and Delta (B. 1.617. 2, India). J. Virus Eradic..

[8] Abu-Raddad L.J., Chemaitelly H., Ayoub H.H., Yassine H.M., Benslimane F.M., Al Khatib H.A., Tang P., Hasan M.R., Coyle P., AlMukdad S. (2021). Severity, Criticality, and Fatality of the Severe Acute Respiratory Syndrome Coronavirus 2 (SARS-CoV-2) Beta Variant. Clin. Infect. Dis..

[9] Faria N.R., Mellan T.A., Whittaker C., Claro I.M., Candido D.D.S., Mishra S., Crispim M.A., Sales F.C., Hawryluk I., McCrone J.T. (2021). Genomics and epidemiology of the P. 1 SARS-CoV-2 lineage in Manaus, Brazil. Science.

[10] Planas D., Veyer D., Baidaliuk A., Staropoli I., Guivel-Benhassine F., Rajah M.M., Planchais C., Porrot F., Robillard N., Puech J. (2021). Reduced sensitivity of SARS-CoV-2 variant Delta to antibody neutralization. Nature.

[11] Dougherty K., Mannell M., Naqvi O., Matson D., Stone J. (2021). SARS-CoV-2 B. 1.617. 2 (Delta) variant COVID-19 outbreak associated with a gymnastics facility—Oklahoma, April–May 2021. Morb. Mortal. Wkly. Rep..

[12] Singh J., Rahman S.A., Ehtesham N.Z., Hira S., Hasnain S.E. (2021). SARS-CoV-2 variants of concern are emerging in India. Nat. Med..

[13] World Health Organization WHO Director-General’s Opening Remarks at the Media Briefing on COVID-19. 30 July 2021. https://www.who.int/director-general/speeches/detail/who-director-general-s-opening-remarks-at-the-media-briefing-on-covid-19-30-july-2021.

[14] Tegally H., Wilkinson E., Giovanetti M., Iranzadeh A., Fonseca V., Giandhari J., Doolabh D., Pillay S., San E.J., Msomi N. (2021). Detection of a SARS-CoV-2 variant of concern in South Africa. Nature.

[15] Gao S.J., Guo H., Luo G. (2022). Omicron variant (B. 1.1. 529) of SARS-CoV-2, a global urgent public health alert!. J. Med. Virol..

[16] Furushima D., Kawano S., Ohno Y., Kakehashi M. (2017). Estimation of the basic reproduction number of novel influenza A (H1N1) pdm09 in elementary schools using the SIR model. Open Nurs. J..

[17] Gaudart J., Ghassani M., Mintsa J., Rachdi M., Waku J., Demongeot J. (2010). Demography and diffusion in epidemics: Malaria and black death spread. Acta Biotheor..

[18] Chowell G., Tariq A., Hyman J.M. (2019). A novel sub-epidemic modeling framework for short-term forecasting epidemic waves. BMC Med..

[19] Yang Z., Zeng Z., Wang K., Wong S.S., Liang W., Zanin M., Liu P., Cao X., Gao Z., Mai Z. (2020). Modified SEIR and AI prediction of the epidemics trend of COVID-19 in China under public health interventions. J. Thoracic Dis..

[20] Li L., Yang Z., Dang Z., Meng C., Huang J., Meng H., Wang D., Chen G., Zhang J., Peng H. (2020). Propagation analysis and prediction of the COVID-19. Infect. Dis. Model..

[21] Caetano C., Morgado M.L., Patrício P., Pereira J.F., Nunes B. (2021). Mathematical Modelling of the Impact of Non-Pharmacological Strategies to Control the COVID-19 Epidemic in Portugal. Mathematics.

[22] Sarkar K., Khajanchi S., Nieto J.J. (2020). Modeling and forecasting the COVID-19 pandemic in India. Chaos Solitons Fractals.

[23] Makhoul M., Ayoub H.H., Chemaitelly H., Seedat S., Mumtaz G.R., Al-Omari S., Abu-Raddad L.J. (2020). Epidemiological impact of SARS-CoV-2 vaccination: Mathematical modeling analyses. Vaccines.

[24] Rodrigues R.F., Silva A.R.D., Fonseca Vieira V.D., Xavier C.R. (2018). Optimization of the choice of individuals to be immunized through the genetic algorithm in the sir model. Proceedings of the International Conference on Computational Science and Its Applications.

[25] Vespignani A., Pastor-Satorras R., Van Mieghem M., Castellano C. (2015). Epidemic processes in complex networks. Rev. Mod. Phys..

[26] Piontti A.P.Y., Gomes M.F.D.C., Samay N., Perra N., Vespignani A. (2014). The infection tree of global epidemics. Netw. Sci..

[27] Nowzari C., Preciado V.M., Pappas G.J. (2015). Optimal resource allocation for control of networked epidemic models. IEEE Trans. Control Netw. Syst..

[28] Nadini M., Rizzo A., Porfiri M. (2018). Epidemic spreading in temporal and adaptive networks with static backbone. IEEE Trans. Netw. Sci. Eng..

[29] Eames K., Keeling M. (2005). Networks and epidemic models. J. R. Soc. Interface.

[30] Lin F., Muthuraman K., Lawley M. (2010). An optimal control theory approach to non-pharmaceutical interventions. BMC Infect. Dis..

[31] Tulu T.W., Tian B., Wu Z. (2017). Modeling the effect of quarantine and vaccination on Ebola disease. Adv. Diff. Equ..

[32] Bar-On Y.M., Goldberg Y., Mandel M., Bodenheimer O., Freedman L., Kalkstein N., Mizrahi B., Alroy-Preis S., Ash N., Milo R. (2021). Protection of BNT162b2 vaccine booster against covid-19 in Israel. N. Engl. J. Med..

[33] Levine-Tiefenbrun M., Yelin I., Katz R., Herzel E., Golan Z., Schreiber L., Wolf T., Nadler V., Ben-Tov A., Kuint J. (2021). Decreased SARS-CoV-2 viral load following vaccination. Nat. Med..

[34] Borse R.H., Shrestha S.S., Fiore A.E., Atkins C.Y., Singleton J.A., Furlow C., Meltzer M.I. (2013). Effects of vaccine program against pandemic influenza A (H1N1) virus, United States, 2009–2010. Emerg. Infect. Dis..

[35] Nguyen C., Carlson J.M. (2016). Optimizing real-time vaccine allocation in a stochastic SIR model. PLoS ONE.

[36] Reis R.F., de Melo Quintela B., de Oliveira Campos J., Gomes J.M., Rocha B.M., Lobosco M., dos Santos R.W. (2020). Characterization of the COVID-19 pandemic and the impact of uncertainties, mitigation strategies, and underreporting of cases in South Korea, Italy, and Brazil. Chaos Solitons Fractals.

[37] Reis R.F., Oliveira R.S., Quintela B.D., Campos J.D.O., Gomes J.M., Rocha B.M., Lobosco M., Santos R.W.D. (2021). The Quixotic Task of Forecasting Peaks of COVID-19: Rather Focus on Forward and Backward Projections. Front. Public Health.

[38] Oliveira R.S., Xavier C.R., da Fonseca Vieira V., Rocha B.M., Reis R.F., de Melo Quintela B., Lobosco M., dos Santos R.W. (2021). How Fast Vaccination Can Control the COVID-19 Pandemic in Brazil?. Proceedings of the Computational Science—ICCS 2021.

[39] Xavier C.R., Oliveira R.S., da Fonseca Vieira V., Rocha B.M., Reis R.F., de Melo Quintela B., Lobosco M., dos Santos R.W. (2022). Timing the race of vaccination, new variants, and relaxing restrictions during COVID-19 pandemic. J. Comput. Sci..

[40] Giordano G., Blanchini F., Bruno R., Colaneri P., Di Filippo A., Di Matteo A., Colaneri M. (2020). Modelling the COVID-19 epidemic and implementation of population-wide interventions in Italy. Nat. Med..

[41] Storn R., Price K. (1997). Differential evolution–a simple and efficient heuristic for global optimization over continuous spaces. J. Glob. Optim..

[42] Lei C.L., Ghosh S., Whittaker D.G., Aboelkassem Y., Beattie K.A., Cantwell C.D., Delhaas T., Houston C., Novaes G.M., Panfilov A.V. (2020). Considering discrepancy when calibrating a mechanistic electrophysiology model. Philos. Trans. R. Soc. A Math. Phys. Eng. Sci..

[43] Worldometers (2020). COVID-19 Coronavirus Pandemic. https://www.worldometers.info/coronavirus/..

[44] Ulloa A.C., Buchan S.A., Daneman N., Brown K.A. (2022). Estimates of SARS-CoV-2 Omicron Variant Severity in Ontario, Canada. JAMA.

[45] Wolter N., Jassat W., Walaza S., Welch R., Moultrie H., Groome M., Amoako D.G., Everatt J., Bhiman J.N., Scheepers C. (2022). Early assessment of the clinical severity of the SARS-CoV-2 omicron variant in South Africa: A data linkage study. Lancet.

[46] Ferguson N., Ghani A., Hinsley W., Volz E. (2021). Report 50: Hospitalisation Risk for Omicron Cases in England.

